# Evaluation of Dexamethasone-Induced Osteoporosis In Vivo Using Zebrafish Scales

**DOI:** 10.3390/ph14060536

**Published:** 2021-06-03

**Authors:** Siripat Chaichit, Takuto Sato, Huiqing Yu, Yu-ki Tanaka, Yasumitsu Ogra, Takamasa Mizoguchi, Motoyuki Itoh

**Affiliations:** 1 Graduate School of Pharmaceutical Sciences, Chiba University, Chiba 260-8675, Japan; siripat_chaichit@cmu.ac.th (S.C.); caya4900@chiba-u.jp (T.S.); yudo.je2j0c@gmail.com (H.Y.); yu-ki.tanaka@chiba-u.jp (Y.-k.T.); ogra@chiba-u.jp (Y.O.); mizoguchi@chiba-u.jp (T.M.); 2 Faculty of Pharmacy, Chiang Mai University, Chiang Mai 50200, Thailand

**Keywords:** zebrafish, scale, cathepsin K, tartrate-resistant acid phosphatase, alkaline phosphatase, glucocorticoid-induced osteoporosis

## Abstract

Glucocorticoid-induced osteoporosis (GIOP) is a major cause of secondary osteoporosis, and the pathogenic mechanisms of GIOP remain to be elucidated. Here, we show a rapid dexamethasone-induced osteoporosis animal model using zebrafish scales. Intraperitoneal injection of dexamethasone over a 5-day period suppressed the regeneration of scales. Furthermore, the circularity of the newly formed regenerated scales was also slightly reduced compared to that of the control group on day 5. The changes in bone-related enzymes, such as cathepsin K, tartrate-resistant acid phosphatase (TRAP) for bone resorption, and alkaline phosphatase (ALP) for bone formation, provide insight into the progression of bone diseases; therefore, we further developed a method to measure the activities of cathepsin K, TRAP, and ALP using zebrafish scales. We found that a lysis buffer with detergent at neutral pH under sonication efficiently helped extract these three enzymes with high activity levels. Interestingly, treatment with a dexamethasone injection produced considerably higher levels of cathepsin K activity and a lower Ca/P ratio than those in the control group, suggesting that dexamethasone increased osteoclast activity, with no significant changes in the activities of TRAP and ALP. Our GIOP model and enzyme assay method could help to design better treatments for GIOP.

## 1. Introduction

Osteoporosis is an age-related bone disease that increases the risk of bone fragility by decreasing the bone strength and density, especially in postmenopausal women and elderly individuals [1]. Aging and hormonal changes lead to an imbalance between bone resorption by osteoclasts and bone formation by osteoblasts [2,3]. An increase in osteoclastic activity and a decrease in osteoblastic activity result in osteoporosis [4]. Therefore, interest in osteoporosis has increased in recent years. A wide variety of osteoporotic models have been generated to support bone research. Glucocorticoid-induced osteoporosis (GIOP) is the most common cause of secondary osteoporosis [5]. Glucocorticoid compounds, such as prednisolone and dexamethasone, exhibit a potent effect on bone metabolism, decreasing the bone mineral density and increasing the risk of bone fracture [6,7]. Previous research has shown that osteoclast activity is increased and osteoblast activity is reduced by glucocorticoid exposure [6,8]. Additionally, it is well known that long-term glucocorticoid exposure can induce bone loss [9]. The pathogenic mechanisms of GIOP remain elusive due to a combination of both local and systemic effects [10]. Therefore, research is needed to develop osteoporosis models for the treatment of GIOP.

Several animal models for GIOP have been reported, including in mice and rats. However, mammalian GIOP models are expensive and time-consuming to generate. Zebrafish have become a popular biomedical model in a wide variety of studies [11,12]. In comparison with other animals, zebrafish possess benefits such as small size, fast development, and rapid genetic modification [12,13]. Moreover, zebrafish display approximately 70% similarity among all genes [14] and 85% similarity among disease genes [15] with humans, which is a considerable advantage of using zebrafish models to provide insight into the mechanisms of human disease [16,17]. Recently, zebrafish GIOP models using scales have been reported. Zebrafish scales contain apatite crystals, which are close to the collagen fibers that build the bone matrix [18,19,20,21], and different cell types, including osteoblasts and osteoclasts, play key roles in their maintenance and regeneration [22]. When a scale is removed, lining cells remaining in the scale pocket differentiate to start forming a new scale [23,24,25]. New scales of zebrafish are formed within two days after scale removal and are fully regrown and mineralized within a month [18,26]. The regeneration paradigm thus offers a model with which to study principles underlying early skeletal development in an adult fish. Many publications have established the zebrafish scale GIOP model, which exhibits an osteoporotic phenotype after treatment with prednisolone and dexamethasone, suggesting the usefulness of this zebrafish scale GIOP model [26,27]. It has been suggested that the zebrafish should be exposed to prednisolone after scale removal for at least 3 weeks to observe the negative effect of prednisolone on regenerated scales [26]. Dexamethasone is a more potent glucocorticoid compound than prednisolone [28]. Therefore, we hypothesized that dexamethasone treatment would induce the osteoporotic phenotype in an early stage of scale regeneration, and it makes the model useful for future drug screening applications.

Therefore, we designed the adult zebrafish scale GIOP model by investigating the activity of bone biomarkers, including the cathepsin K, TRAP, and ALP enzymes [29]. Cathepsin K and TRAP are well-known bone resorption biomarkers produced from mature osteoclasts and are used as indicators of osteoclast activity [29,30]. Cathepsin K is a cysteine protease highly expressed in osteoclasts and is responsible for cleaving and degrading the organic matrix in an acidic pH environment [18]. This enzyme is also found in various tissues, such as chondrocytes, synovial fibroblasts, and macrophages [31,32,33]. In zebrafish scales, cathepsin K is found in multinucleated osteoclasts in the marginal regions of adult scales [34,35,36]. TRAP is an acid phosphatase enzyme involved in bone matrix degradation at an optimum low pH [37]. TRAP has been widely used as a bone resorption marker correlated with the number of osteoclasts [29]. ALP is a biomarker related to bone formation that promotes mineralization [38]. To measure enzyme activity, cathepsin K [39] and TRAP enzymes [40] require an acidic environment for their activity, whereas the optimum ALP activity is observed at an alkaline pH [41]. Several studies have shown that the activities of TRAP and ALP can be separately measured using fish scales incubated in different reaction buffers (acidic and alkaline buffers for TRAP and ALP assays, respectively) without detergent [23,25]. However, high individual variations among scales from the same zebrafish were observed [23,26,42]. The determination of enzyme activities from the same extracts could reduce individual variations and be more accurate.

In this study, we aimed to establish a dexamethasone-induced GIOP zebrafish model in a short period (5 days) using zebrafish scales and evaluated cathepsin K, TRAP, and ALP activity in the same sample by our developed method.

## 2. Results

### 2.1. Intraperitoneal Dexamethasone Injection Reduced the Regeneration of Zebrafish Scales

**Figure 1:**
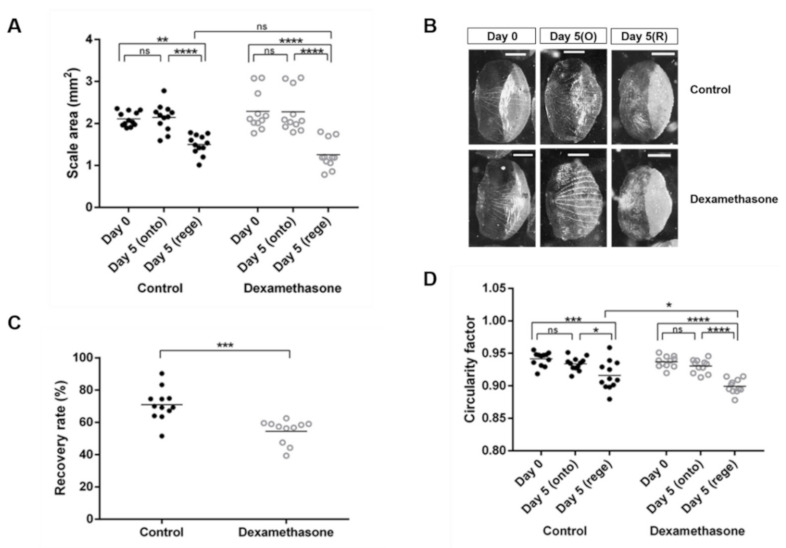
Morphometric analyses of zebrafish scales on day 0, ontogenetic scales on day 5, and regenerated scales on day 5 from the control (black dots) and dexamethasone-treated groups (open dots). Each dot represents an average value of each fish: (**A**) Scale area (mm^2^), (**B**) Representative scales from control and dexamethasone group on day 0 and day 5 (scale bars = 0.5 mm), (**C**) Percentage of the recovery rate of scale regeneration, (**D**) Circularity factor (4πA/P^2^; where A is the surface area and P is the perimeter). Interactions between groups were assessed with two-way ANOVA with Tukey’s multiple comparisons test. Data are shown as the mean ± SEM (**** *p* ≤ 0.0001; *** *p* ≤ 0.001; ** *p* ≤ 0.01; * *p* ≤ 0.05; ns: not significant). Error bars indicate the standard error of the mean.

To evaluate zebrafish scales as an in vivo model for dexamethasone-induced osteoporosis, we examined the size and shape of both the ontogenetic and regenerated scales from fish with or without dexamethasone treatment on day 0 and day 5. The term ‘ontogenetic scales’ was used to describe the original scale that grows during ontogeny, while the ‘regenerated scales’ referred to the newly formed scales after regeneration. The surface area of the ontogenetic scales collected on day 5 was not significantly different from that of the original scale at day 0, regardless of dexamethasone treatment (Figure 1A). The average surface area of the regenerated scales was smaller than that of the original scales (day 0), but the difference between the control and dexamethasone groups was not significant (Figure 1A). Images of representative scales from each group were captured (Figure 1B). Taking into consideration the individual differences, the recovery rate of scales of each fish after regeneration was further calculated. The recovery rate of the control group was significantly higher than that of the dexamethasone group (Figure 1B), suggesting that dexamethasone tended to suppress the regeneration of scales. Furthermore, the circularity of the regenerated scales was also slightly reduced compared to that of the original scales, and the regenerated scales from the dexamethasone-treated group on day 5 showed a lower circularity factor compared to those from the control group (Figure 1C).

### 2.2. Lysate from Scales Is Beneficial for the Measurement of Cathepsin K, TRAP, and ALP Activities

**Figure 2:**
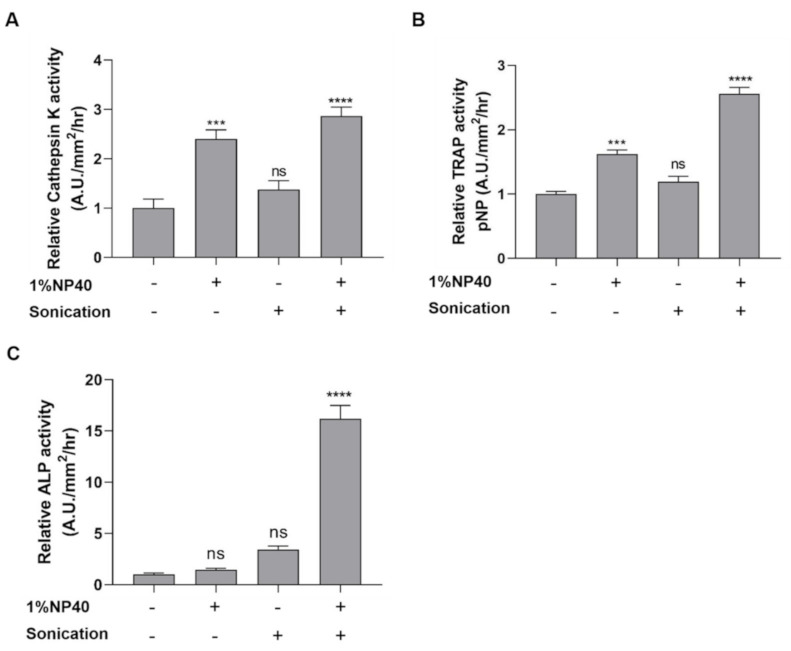
Effect of detergent (1% NP-40) and sonication on enzymatic activities relative to the control. The scale samples lysed in the buffer in the presence (+) or absence (−) of 1%NP40 and sonication. (**A**) Cathepsin K activity, (**B**) TRAP activity, and (**C**) ALP activity. Interactions between groups were assessed with one-way ANOVA. The significant statistical analyses were analyzed with Tukey’s multiple comparison test. Data are shown as the mean ± SEM (**** *p* ≤ 0.0001; *** *p* ≤ 0.001; ns: not significant). Error bars indicate the standard error of the mean.

To evaluate the enzymatic activities of the bone-related enzymes cathepsin K, TRAP, and ALP in scales, we designed and developed a method to improve enzyme extractability by the use of a nonionic detergent (1% NP-40) and a sonication process in a basal lysis buffer (10 mM Tris pH 7.5, 150 mM NaCl, 1 mM EDTA). The scale samples lysed in the buffer containing 1% NP-40 showed stronger cathepsin K activity than did those lysed in the buffer without detergent, while the cathepsin K activity obtained by sonication was comparable to that obtained without sonication (Figure 2A). Interestingly, the results obtained using a combination of 1% NP-40 and sonication suggested considerably high cathepsin K levels in the extract (Figure 2A). Similar trends were also observed in the TRAP assay (Figure 2B). Higher levels of TRAP activity were obtained in the extracts using 1% NP-40 and the combination of detergent and sonication, while sonication showed a lesser effect on TRAP extraction. However, the use of only detergent or sonication showed less capability to extract high amounts of ALP enzymes from the scale extract compared to the use of the combination of the two techniques (Figure 2C). The utility of 1% NP-40 and sonication demonstrated a considerable increase in ALP activity in the lysate. According to these data, we designed the extraction method using 1% NP-40 and sonication.

**Figure 3:**
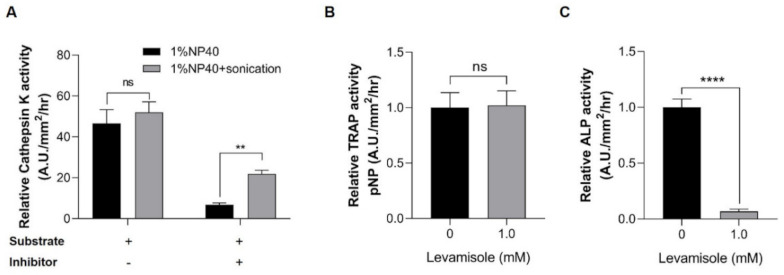
Inhibitor specificity in enzyme assays. (**A**) Cathepsin K activity. The lysates extracted under 1% NP-40 only (black bar) and 1% NP-40+sonication (gray bar) conditions were used in the cathepsin K assay. The lysates were incubated with a substrate and the presence (+) or absence (−) of cathepsin K inhibitor. The enzymatic activity was calculated as the ratio between the fluorescence values of the lysate and background (relative to 1), (**B**) TRAP activity, and (**C**) ALP activity. Lysates extracted under 1% NP-40 and sonication conditions were incubated with an acidic buffer and an alkaline buffer containing pNPP and 1 mM levamisole (an ALP inhibitor) to measure the activities of TRAP and ALP, respectively. These activities were determined using the UV spectroscopy method (λ = 405 nm). Values are presented as the mean ± SEM. Significant differences were calculated by Student’s *t*-test, with **** *p* ≤ 0.0001; ** *p* ≤ 0.01; ns: not significant. Error bars indicate the standard error of the mean.

We then evaluated the inhibitor specificity in lysate samples extracted from our method. The cathepsin K activities from samples lysed in buffer containing 1% NP-40 were comparable to those from 1% NP-40 and sonication (Figure 3A). The specific activity of the enzyme was further confirmed using a cathepsin K inhibitor (Z-Leu-Leu-Leu-H). The inhibitory activity of the sample lysed in 1% NP-40 was more potent than those lysed under the combination of the two conditions (Figure 3A), suggesting that measurement of cathepsin K activity in a buffer containing 1% NP-40 is more suitable than that using 1% NP-40 and sonication.

Additionally, TRAP and ALP analyses used the same lysate and substrate, and the inhibitor study was further performed using levamisole, which is a well-known endogenous ALP inhibitor. The samples lysed under 1% NP-40 and sonication conditions were pretreated with levamisole before incubation in the individual reaction buffers. The relative TRAP activity of the control group was comparable to that of the levamisole-treated group (Figure 3B). Conversely, the control group exhibited a higher ALP activity than that of the levamisole pretreatment group (Figure 3C). Given that ALP activity was significantly decreased in the levamisole-treated group, our method enabled the specific detection of the activities of ALP and TRAP in scale lysates. Regarding these experiments, we reasoned that samples for TRAP and ALP assays should be prepared under 1% NP-40 and sonication conditions, and the inhibitor study further confirmed the specificity of these enzymes.

**Figure 4:**
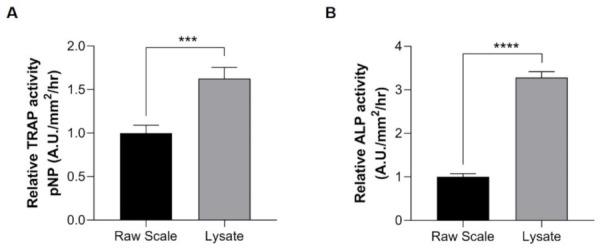
Determination of enzyme activities using raw scales (black bar) and lysate (gray bar). (**A**) TRAP activity and (**B**) ALP activity. Lysate samples (gray bar) exhibit high activity levels compared with raw scale samples (black bar). Comparisons between the two groups were performed by Student’s *t*-test. The comparison of the methods was tested by the unpaired *t*-test method. Data are shown as the mean ± SEM (**** *p* ≤ 0.0001; *** *p* ≤ 0.001). Error bars indicate the standard error of the mean.

We further used scale lysates to investigate the effectiveness of our method. When comparing our method with previously reported methods using raw scales [27,36,43], the activities of TRAP and ALP were significantly higher in our method using lysate samples (Figure 4A,B). Our method showed an approximately 1.6-fold increase in TRAP activity levels compared with the reported raw scale method (Figure 4A). Interestingly, our method also exhibited a dramatic increase (~3-fold) in ALP activity compared with raw scale samples (Figure 4B). These experiments indicated the effectiveness of our method with the use of scale lysates.

**Figure 5:**
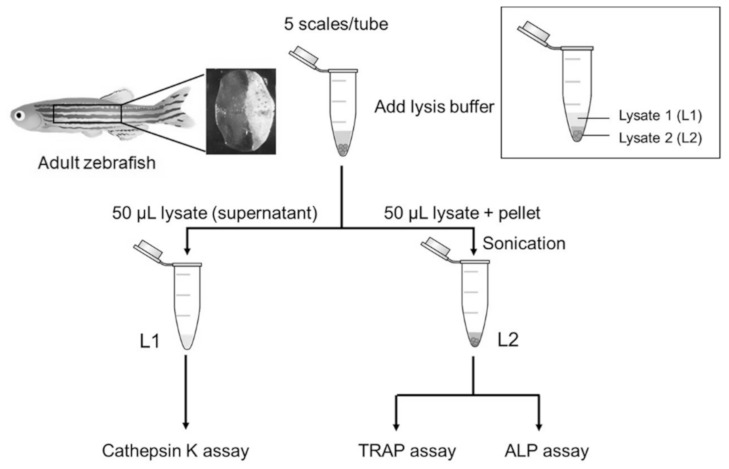
The schematic diagram of the scale lysis method. Five fish scales were pooled in a microcentrifuge tube. When starting an experiment, the scales were incubated in lysis buffer and then centrifuged at 17,400× *g*. The first half of the lysate (supernatant) was collected for cathepsin K assay. The remaining lysate was sonicated and then used for TRAP and ALP assays.

To better understand the mechanisms underlying the dexamethasone-induced reduction in scale regeneration, we further used our developed assay to evaluate the activity of enzymes involved in bone remodeling from the same sample. In total, five zebrafish scales were pooled and incubated with lysis buffer. After centrifugation, the solution was separated into two parts, Lysate 1 (L1) and Lysate 2 (L2), as shown in Figure 5. L1 was the first half of the solution (supernatant), and L2 was the second half of the solution (lysate and pellet), which was prepared by sonication to extract the remaining proteins. Then, we examined the enzymatic activities of cathepsin K, TRAP, and ALP in the two lysates.

### 2.3. Effect of Dexamethasone on Cathepsin K, TRAP and ALP Activities

**Figure 6:**
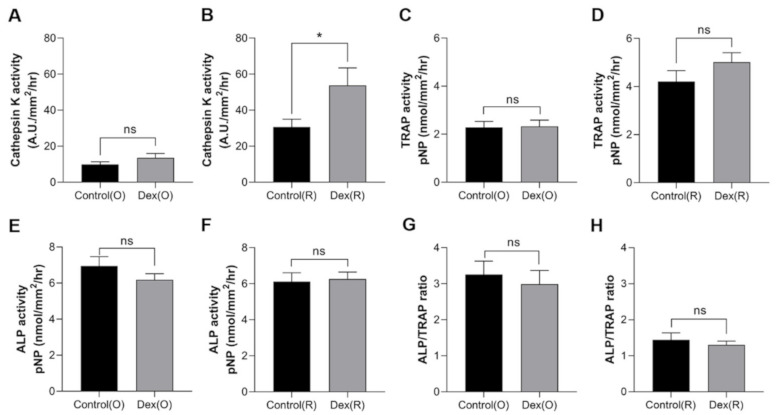
Enzymatic activities of scale lysates from the control (black bar) and dexamethasone-treated groups (gray bar) on day 5 of the experiment. Cathepsin K activity of (**A**) ontogenetic scales and (**B**) regenerated scales, TRAP activity of (**C**) ontogenetic scales and (**D**) regenerated scales, ALP activity of (**E**) ontogenetic scales and (**F**) regenerated scales, and ALP/TRAP ratio of (**G**) ontogenetic scales and (**H**) regenerated scales. Comparisons between groups were assessed with Student’s *t*-test. Data are shown as the mean ± SEM (* *p* ≤ 0.05; ns: not significant). Error bars indicate the standard error of the mean.

We next examined the effects of dexamethasone on cathepsin K, TRAP, and ALP activity after a 5-day period, as described previously. The cathepsin K activity of ontogenic scales in the dexamethasone-treated groups on day 5 tended to be higher than that of ontogenetic scales in the control group, although the difference was not statistically significant (Figure 6A). In contrast, the regenerated scales from the dexamethasone-treated group showed significantly higher levels of cathepsin K activity compared to those from the control group (Figure 6B). The TRAP activity of ontogenetic or regenerated scales was not significantly different between the control and dexamethasone-treated groups (Figure 6C,D). The ALP activity was not significantly different among all groups (Figure 6E,F). We then examined the ratio of the activities of ALP to TRAP (Figure 6G,H). The ALP/TRAP ratio was not altered either in the control or regenerated scales with dexamethasone treatment. This finding may be interpreted as evidence that the ratio of osteoblasts to osteoclasts in scales did not change at day 5 after dexamethasone treatment.

### 2.4. Effect of Dexamethasone on the Mineral Composition of Zebrafish Scales

**Figure 7:**
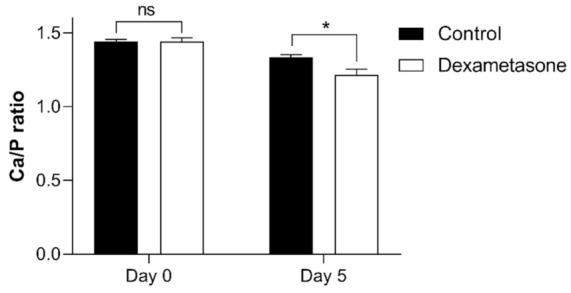
Ca/P ratio of zebrafish scales on day 0 and day 5 of the experiment from the control (black bar) and dexamethasone groups (white bar). Ten scales from each were collected and measured the calcium and phosphorus levels using ICP-MS. Values are presented as the mean ± SEM using Student’s *t*-test, with * *p* ≤ 0.05; ns: not significant. Error bars indicate the standard error of the mean.

To further evaluate the effect of dexamethasone on scales, the calcium and phosphorus contents were measured in the original scales and regenerated scales (Figure 7). The calcium-to-phosphorus (Ca/P) ratio of the regenerated scales (day 5) exhibited low values compared to those of the original scales from both groups. No differences in the Ca/P ratio were observed in the original scales between the control and dexamethasone-treated groups. However, we observed an effect of dexamethasone on the regenerated scales at 5 days of regeneration, with a Ca/P value smaller than that of the control group.

## 3. Discussion

### 3.1. Dexamethasone-Induced Osteoporosis Using Zebrafish Scales

In this study, dexamethasone treatment increased osteoclast activity (i.e., cathepsin K activity) but not osteoblast activity (i.e., ALP activity). This observation is supported by similar findings in scales treated with prednisolone, in which the expression of osteoblast markers, including ALP, was not increased [26]. Furthermore, it has been suggested that glucocorticoids cause early and transient increases in bone resorption and decrease osteoblastogenesis during long-term treatment [44]. In contrast, Sato et al. showed that osteoclast activity was suppressed by bath treatment of dexamethasone at an early stage of scale regeneration [45]. This difference in osteoclast activity from our study should warrant further investigation.

Typically, a ratio of calcium phosphate of approximately 1.67 indicates pure hydroxyapatite in the bone [46]. After treatment, all the regenerated scales showed low Ca/P ratios compared to ontogenetic and original scales, suggesting changes in the crystalline phase. The decrease in the Ca/P ratio in the zebrafish scales is correlated with the increase in osteoclastic activity, which was reported in previous studies [23,26]. This situation would lead to unstable minerals easily dissolved by osteoclasts during scale mineralization [26].

Accordingly, our dexamethasone-induced model recapitulated the early GIOP phenotype (i.e., increased osteoclast activity) but did not reduce osteoblast cell activity well. Our experiment used dexamethasone, which is a more potent glucocorticoid than prednisolone, to induce early-stage osteoporotic characteristics in zebrafish [45,47]. Although cathepsin K activity was increased by dexamethasone treatment, we found that the ratio of the activities of ALP/TRAP remained unchanged. Therefore, more research is needed to clarify the mechanisms underlying the increased osteoclast activity at the cellular and molecular level. Moreover, our model induced the osteoporosis phenotype in regenerated scales by intraperitoneal (IP) injections of dexamethasone. Most zebrafish GIOP models were developed by bath treatment of prednisolone or dexamethasone [26,27,45]. Our IP injection protocol requires a smaller amount of dexamethasone compared to bath treatment and that used for rainbow trout [48], and thus it could serve to improve the performance of drug screening. Taken together, these results show that our scale model allows the rapid evaluation of GIOP-induced osteoclast activity in 5 days, and it will be useful for pharmacological screening to find new anti-osteoporotic compounds.

### 3.2. Our Developed Assay Allows the Effective Measurement of the Activities of Three Bone-Related Enzymes

Our study used cathepsin K and TRAP as bone-degradation markers and ALP as a bone-formation marker. However, TRAP and cathepsin K are not confined to osteoclasts [49,50], similarly to ALP, which is not restricted to osteoblasts [51,52]. Nonetheless, they have been used extensively as bone-related biomarkers to help in osteoporosis assessments in the early stage [53]. Cathepsin K has gained increased interest due to its role in bone resorption and is considered a novel target for osteoporosis treatment [54]. Its activity has been measured in vitro and in vivo in several models, including humans, to discover new agents for treating osteoporosis and other disorders [55,56,57]. However, procedures for the isolation and generation of osteoclasts from rodents or humans are expensive and time-consuming [58]. Given the high demand in the bone research field, our method provides an effective way to measure bone metabolism using adult zebrafish scales [34].

In our study, we established a method to extract cathepsin K, as well as ALP and TRAP, from zebrafish scales using a Tris-based buffer containing detergent (1% NP-40). Compared with previously reported methods, our method exhibits a beneficial capability to determine the activities of three bone-related enzymes using the same samples. It allows simple and reproducible analyses with reduced variation originating from the individual scales. Previous experiments used raw scales to analyze the activities of TRAP and ALP [36,59]. To measure the activity of each enzyme under different pH conditions, the samples were separately prepared, and thus, a large number of scales were needed for each assay due to high variation in each scale. Our method uses the same samples for the three enzymes, but different lysates because of the distinct effects of sonication on each enzyme. Sonication helps extract TRAP and ALP but not cathepsin K, although the TRAP enzyme is known to be secreted from lysosomes, as is cathepsin K, and ALP is observed on the cell surface and in matrix vesicles [60]. The effect of sonication on ALP extraction was supported by the use of levamisole as an ALP inhibitor. Consistent with previous studies, levamisole exhibited the capability to inhibit ALP activity in in vitro studies [61,62]. Our results confirm that high ALP activity levels resulted from the efficient extraction method, especially after using the sonication process during enzyme extraction. In the case of cathepsin K activity, sonication even reduced the enzymatic specificity, as judged by the inhibitor treatment results. Therefore, a future evaluation may require careful consideration of the enzymatic activities produced by our method, but our findings could help clarify the mechanism underlying the regulation of the activity of these enzymes.

## 4. Materials and Methods

### 4.1. Zebrafish Husbandry

Adult zebrafish were purchased from Masuko Co., Ltd. (Japan). The fish were separated and kept in a 1.3 L tank (Meito System) under a normal light/dark cycle of 14 h/10 h at 28 °C. The fish were fed twice daily with Otohime B2 (Marubeni Nisshin Feed, Tokyo, Japan) and freshly hatched *Artemia* cysts (A&A Marine LLC, Murray, UT, USA) and kept for one week before starting experiments.

### 4.2. Dexamethasone Treatment and Sample Collection

Dexamethasone 21-phosphate was purchased from Wako Inc. (Tokyo, Japan). The concentration of dexamethasone solution used was 30 μg per g fish body weight [48]. The methods of dexamethasone injection were designed based on our preliminary studies (adapted from the previous study [26]) to obtain potent osteoclastic activity, especially during scale regeneration. A 0.9% *w*/*v* sodium chloride (NaCl) solution was used as a vehicle for dissolving dexamethasone and for treatment in the control group. On the first day of the experiment (day 0), the original scales were removed. Dexamethasone (9.68 mM) and NaCl solution were injected through the peritoneum of the fish in the treated group and control group, respectively. The injections were given on days 0, 2, and 4 in both groups. At the end of the experiment (day 5), the regenerated scales and ontogenetic scales were harvested and collected for further experiments.

### 4.3. Morphometric Analysis

Scale images were captured using AxioCam MRc (Carl Zeiss, Oberkochen, Germany) on a Leica microscope, and the surface area and perimeter were measured by the ImageJ program [63]. The scale surface area was calculated using the total perimeter of the raw scale. The recovery rate was estimated by the percentage of scale surface area on day 5 compared to that on day 0. The circularity factor was calculated as a function of the surface area and perimeter ($\frac{4\pi A}{P^{2}}$, where A is the surface area of the scale and *P* is the perimeter of the scale).

### 4.4. Scale Lysis Method

Prior to scale harvesting, the zebrafish were anesthetized using 0.016% *w*/*v* tricaine methanesulfonate (FUJIFILM Wako Chemicals, Osaka, Japan) solution [64]. Scales were gently removed from the left flank of the fish. Five scales were pooled in PBS solution (137 mM NaCl, 2.7 mM KCl, 10 mM Na_2_HPO_4_, 1.8 mM KH_2_PO_4_, pH 7.3) in a 1.5 mL microcentrifuge tube. When starting an experiment, the PBS solution was removed, and 100 μL of lysis buffer (10 mM Tris-HCl, 150 mM NaCl, 1 mM EDTA, 1% *v*/*v* NP-40, pH 7.5) was added to lyse the scales. The scales were incubated in lysis buffer for 40 min on ice and then centrifuged at 17,400× *g* for 15 min at 4 °C. The first half of the lysate was collected for cathepsin K activity measurement. The remaining lysate was sonicated for a total time of 12.5 min. Then, this solution was used to measure TRAP and ALP activity.

### 4.5. Biochemical Cathepsin K, TRAP, and ALP Scale Assays

A method for determining cathepsin K activity was adapted from Bossard et al. [39]. Ten microliters of lysate was incubated with assay buffer. Activation of the enzyme was achieved by the addition of 20 μL of 10 μM Z-Leu-Arg-MCA (Peptide Institute, Inc., Osaka, Japan) as a fluorogenic substrate [39]. The reaction was incubated for 1 h at 28 °C. The assay buffer for this experiment was 100 mM sodium acetate buffer (pH 5.5) containing 1 mM EDTA and 1 mM dithiothreitol (DTT). The fluorescence intensity was determined using a SpectraMax i3 Multimode Detection Platform (Molecular Devices LLC, Sunnyvale, CA, USA). The excitation and emission wavelengths were set at 380 and 460 nm, respectively.

To determine the cathepsin K activity of each assay, the following formula was used:

$$\mathrm{Cathepsin}\;K\;\mathrm{activity}\left(A.U./{\mathrm{mm}}^{2}/\mathrm{hr}\right)=\frac{\left(\mathrm{FL}\;\mathrm{of}\;\mathrm{treated}\;\mathrm{sample}-\mathrm{FL}\;\mathrm{of}\;\mathrm{blank}\right)}{\left(\mathrm{FL}\;\mathrm{of}\;\mathrm{background}-\mathrm{FL}\;\mathrm{of}\;\mathrm{blank}\right)*\mathrm{surface}\;\mathrm{area}\left({\mathrm{mm}}^{2}\right)*\mathrm{incubation}\;\mathrm{time}(\mathrm{hr})}$$

where FL is fluorescence intensity.

$$\mathrm{Relative}\;\mathrm{cathepsin}\;K\;\mathrm{activity}\left(A.U./{\mathrm{mm}}^{2}/\mathrm{hr}\right)=\frac{\mathrm{Cathepsin}\;K\;\mathrm{activity}\;\mathrm{of}\;\mathrm{treated}\;\mathrm{group}}{\mathrm{Cathepsin}\;K\;\mathrm{activity}\;\mathrm{of}\;\mathrm{control}}$$

The method for TRAP activity measurement was performed according to a previously described protocol [43]. Ten microliters of lysate was used for analysis in 100 μL of reaction buffer and 100 mM sodium acetate buffer at pH 5.3 containing 20 mM tartrate. The total enzymatic reaction was activated by adding 20 mM p-nitrophenylphosphoric acid (pNPP), and the mixture was then incubated for 1 h at 28 °C. Subsequently, the reaction was stopped by adding 50 μL of 3 M sodium hydroxide. The molecular absorbance was determined using a FilterMax F5 (Molecular Devices LLC, Sunnyvale, CA, USA) at a wavelength of 405 nm.

The method for measuring ALP activity followed a previous protocol with some modifications [65]. Ten microliters of lysate was used for analysis in 100 μL of reaction buffer (100 mM Tris-HCl buffer, pH 9.5, containing 20 mM MgCl_2_ and 0.1 mM ZnCl_2_). The total enzymatic reaction was activated by adding 20 mM pNPP, and the mixture was then incubated for 1 h at 28 °C. Subsequently, the reaction was stopped by adding 50 μL of 3 M sodium hydroxide (NaOH). The molecular absorbance was determined using a FilterMax F5 (Molecular Devices LLC, Sunnyvale, CA, USA) at a wavelength of 405 nm.

TRAP and ALP activities were indirectly calculated from the hydrolysis of pNPP to p-nitrophenol (pNP), which is a colored compound. The concentration of pNP was used as a standard curve, and the following formula was used:

$$\mathrm{pNP}\left(\mathrm{nmol}/{\mathrm{mm}}^{2}/\mathrm{hr}\right)=\frac{\mathrm{Concentration}\;\mathrm{of}\;\mathrm{pNP}(\mathrm{nM})\;*\;\mathrm{Total}\;\mathrm{volume}\;(\text{µ}L)}{{\mathrm{Total}\;\mathrm{surface}\;\mathrm{area}\;(\mathrm{mm}}^{2})\;*\;\mathrm{incubation}\;\mathrm{time}\;(\mathrm{hr})}$$

### 4.6. Inhibitor Specificity of Cathepsin K, TRAP and ALP in Zebrafish Scale Lysates

For the inhibitor specificity of cathepsin K, 10 μL of lysate extracted in buffer containing 1% NP-40 was incubated with 10 μL of 10 μM Z-Leu-Leu-Leu-H (Peptide Institute, Inc.), which is a cathepsin K inhibitor, and the assay buffer was incubated for 40 min on ice before adding the cathepsin K substrate, followed by the method for cathepsin K measurement described above.

For the TRAP and ALP assays, the scale lysates extracted under 1% NP-40 sonication conditions were pretreated with 1 mM levamisole for 40 min at 28 °C. The samples were then incubated with an individual reaction buffer containing pNPP solution for 1 h at 28 °C. The molecular absorbance was determined, followed by TRAP and ALP assays, as mentioned earlier.

### 4.7. Mineral Content Determination Using the Inductively Coupled Plasma Mass Spectrometry (ICP-MS) Method

Ten ontogenetic and regenerated scales each were collected and lysed with 100 μL 61% nitric acid (HNO_3_) on a hot plate at 230 °C for 5 h. Fifty microliters of the sample was diluted in 5 mL of 1% diluted HNO_3_ and kept at 4 °C until the determination mentioned below. Mineral contents such as calcium and phosphorus elements were determined using ICP-MS (Agilent 8800 Triple Quadrupole ICP-QQQ, Agilent Technologies, Hachioji, Japan). Then, the Ca/P molar ratios were calculated. This method was adapted from previous protocols [23,26].

### 4.8. Statistical Analysis

All statistical analyses were performed using GraphPad Prism Version 6 for Windows (GraphPad Software, San Diego, CA, USA). One-way and two-way ANOVA with Tukey’s multiple comparisons test were used to determine the interaction between groups in several experiments. Comparisons between two groups were performed by Student’s *t*-test. The comparison of methods was tested by the paired *t*-test method. Data are shown as the mean ± SEM (**** *p* ≤ 0.0001, *** *p* ≤ 0.001, ** *p* ≤ 0.01, and * *p* ≤ 0.05).

## 5. Conclusions

The need for high-throughput screening has arisen from the demands of research activities. Our study provides a straightforward bone assessment method using adult zebrafish scales to measure the activities of cathepsin K, TRAP, and ALP. Furthermore, an increase in osteoclastic activity was observed on day 5 of regeneration after dexamethasone exposure, as indicated by the high levels of cathepsin K and lower Ca/P ratio. To summarize, our zebrafish scale GIOP model and the scale lysis method offer useful tools for bone research, especially in medium-throughput drug screening.

## Data Availability

Data is contained within the article.

## Abbreviations

TRAP; tartrate-resistant acid phosphatase, ALP; alkaline phosphatase
